# TULIP2: An Improved Method for the Identification of Ubiquitin E3-Specific Targets

**DOI:** 10.3389/fchem.2019.00802

**Published:** 2019-11-26

**Authors:** Daniel Salas-Lloret, Giulia Agabitini, Román González-Prieto

**Affiliations:** González-Prieto Laboratory, Department of Cell and Chemical Biology, Leiden University Medical Center (LUMC), Leiden, Netherlands

**Keywords:** ubiquitin, E3 enzymes, proteomics, post-translational modifications, mass spectrometry

## Abstract

Protein modification by Ubiquitin or Ubiquitin-like modifiers is mediated by an enzyme cascade composed of E1, E2, and E3 enzymes. E1s, or ubiquitin-activating enzymes, perform ubiquitin activation. Next, ubiquitin is transferred to ubiquitin-conjugating enzymes or E2s. Finally, ubiquitin ligases or E3s catalyze the transfer of ubiquitin to the acceptor proteins. E3 enzymes are responsible for determining the substrate specificity. Determining which E3 enzyme maps to which substrate is a major challenge that is greatly facilitated by the TULIP2 methodology. TULIP2 methodology is fast, precise, and cost-effective. Compared to the previous TULIP methodology protocol, TULIP2 methodology achieves a more than 50-fold improvement in the purification yield and two orders of magnitude improvement in the signal-to-background ratio after label free quantification by mass spectrometry analysis. The method includes the generation of TULIP2 cell lines, subsequent purification of TULIP2 conjugates, preparation, and analysis of samples by mass spectrometry.

## Introduction

The development of liquid chromatography tandem mass spectrometry (LC-MS/MS)-based proteomics technology has boomed in the past years, and, recently, a new strategy termed UbiSite, enabled the identification of around 63,000 unique sites for ubiquitination at endogenous levels of more than 10,000 proteins, including N-terminal ubiquitination (Akimov et al., 2018). The identification of additional ubiquitination sites seems to be a matter of repeating the UbiSite strategy with samples from different sources.

Determining which E3 enzyme is responsible for modifying which substrate is challenging. Different strategies have been proposed for identification of specific E3 substrates. Many of these strategies are based on indirect evidence. For example, investigating differences in the ubiquitin proteome upon overexpression or depletion of a specific E3 (Song et al., 2011; Sarraf et al., 2013; Thompson et al., 2014). Proteins that are enriched or depleted, respectively, in their ubiquitination levels are considered putative ubiquitination substrates for the specific E3 under investigation. However, the complexity of full ubiquitin proteomes is high (Akimov et al., 2018), and low abundant ubiquitination targets might be missed. Furthermore, results obtained from overexpression-based screens might be due to overexpression artifacts. In the case of the knock down-based screens, E3 ligases can be redundant on their targets, and some targets might be missed because their ubiquitination is still performed by another E3 enzyme. E3 enzyme cascades exist, and the absence of a specific ubiquitinated protein might be a result of an epistatic effect. Thus, every target has to be very carefully verified. As a consequence, indirect approaches are unable to find E3-specific substrates in a reliable manner.

**Figure 1:**
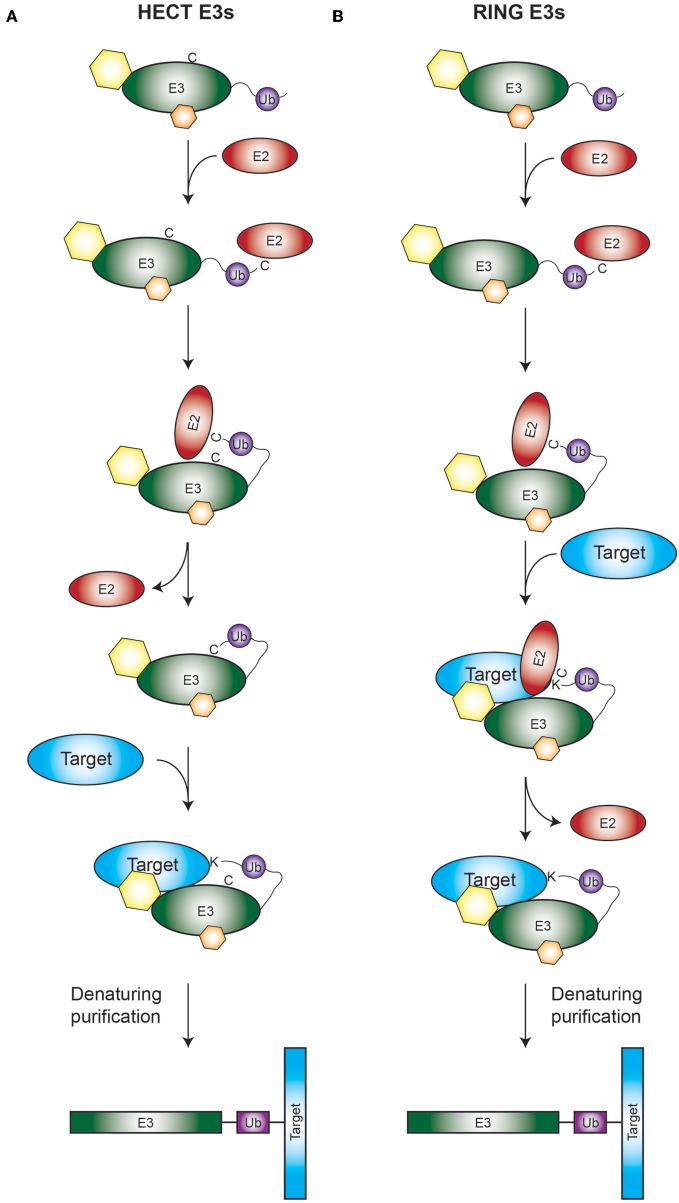
Rationale of the TULIP2 methodology. Rationale is depicted for both HECT **(A)** and RING **(B)** E3 enzymes. **(A)** Activated ubiquitin linearly fused to a HECT E3 of interest will be conjugated to its respective E2 and transferred from the catalytic cysteine of the E2 to the catalytic cysteine of the HECT E3. Next ubiquitin will be transferred from the catalytic cysteine of the E3 to the acceptor lysine of the E3-target protein. Ubiquitination target will remain covalently bound to the E3, enabling the purification of the E3 together with the target protein. **(B)** Similar to A, but in this case the RING E3 catalyzes the transfer of its attached ubiquitin directly from the catalytic cysteine of its respective E2 to the ubiquitination target. Hexagons represent non-covalent interactors of the E3s.

A proposed direct approach is the employment of ubiquitin-activated interaction traps, UBAITs (O'Connor et al., 2015), which work both for Really Interesting New Gene (RING) and Homologous to E6AP C-Terminus (HECT)-type E3 enzymes. The UBAIT approach is based on the utilization of E3 enzyme-ubiquitin fusions. The rationale behind this technique is that, if a linear fusion between a specific E3 and ubiquitin is made, the E3 will be prone to use this ubiquitin to conjugate it to its ubiquitination target. Therefore, the E3 will remain covalently bound to its target after ubiquitination, which allows the later purification of the E3 together with its ubiquitination target. Enabling subsequent identification by LC-MS/MS analysis (Figure 1). The main pitfall of the UBait approach is that the purification of the conjugates is based on epitope-antibody interaction, which excludes the possibility of using denaturing buffers. This disadvantage makes it difficult to distinguish between ubiquitination targets and other potential strong interactors of the E3s. Additionally, it is based on overexpression of the constructs, so the occurrence of overexpression-derived artifacts is a possibility.

Nevertheless, using the UBAIT as a base, we optimized and designed a systematic methodology which we termed Targets of Ubiquitin Ligases Identified by Proteomics (TULIP) (Kumar et al., 2017). TULIP methodology employs 10xHIS nickel-based purification, which allows the use of harsh denaturing buffers, solving the drawback of being unable to distinguish between ubiquitination targets and interactors of the E3. Moreover, TULIP methodology is lentiviral based, employing an all-in-one doxycycline-ON system followed by Gateway® cloning cassette and puromycin as selection marker for infected cells. TULIP methodology enables the generation of stable-inducible cell lines where the expression levels can be titrated to near-to-endogenous levels, minimizing the probability of obtaining results due to overexpression. The C-terminal GlyGly motif of ubiquitin is required for conjugation to a target. TULIP plasmids where ubiquitin lacks the C-terminal GlyGly motif (TULIP-ΔGG) are also available as negative controls. Furthermore, catalytically-dead mutants of the E3 enzymes are used as an additional negative control.

In this article, we describe an improved version of the TULIP methodology (Kumar et al., 2017), which we have termed TULIP2. TULIP2 introduces an extra 10xHIS N-terminal tag preceding the Gateway® cloning cassette. The addition of the extra 10xHIS tag results in an average improvement of more than 50 times in terms of purification efficiency of the TULIP conjugates and an improvement of two orders of magnitude in the signal-to-background ratio after mass spectrometry and Label Free Quantification (LFQ) analysis for the SUMO-Targeted Ubiquitin Ligase (STUbL) RNF4.

## Methods

### Materials, Reagents, and Antibodies

Dulbecco's modified Eagle's medium, penicillin/streptomycin solution, trypsin-EDTA solution were acquired from Life Technologies (Carlsbad, CA, USA). Fetal bovine serum was from Biowest (Nuaillé, France). Di-sodium hydrogen phosphate dihydrate (Na_2_HPO_4_•2H_2_O) was from VWR chemicals (Radnor, PA, USA). Sodium dihydrogen phosphate monohydrate (NaH_2_PO_4_•H_2_O), sodium chloride, trifluoroacetic acid, tween-20, puromycin dihydrochloride and imidazole were acquired from Merck (Darmstadt, Germany). Sodium dodecyl sulfate (SDS), MOPS running buffer and Guanidine hydrochloride 99.5+% were acquired from Thermo Fisher Scientific (Waltham, MA, USA). Nonidet P-40, formic acid (LC-MS grade), methanol (chromasol HPLC), acetonitrile (HPLC grade), MG132 (Z-leu-leu-leu-al) ≥90% HPLC, doxycycline, ponceau-S, polyethylenimine (PEI), urea, ammonium bicarbonate, polybrene, β-mercaptoethanol, and Triton X-100 were from Sigma Aldrich (St. Louis, MO, USA). C18 (Octadecyl) matrix for STAGE-tips was from Bioanalytical Technologies 3M Company (St. Paul, MN, USA). Phosphate-Buffered Saline (PBS) was from Fresenius Kabi (Bad Homburg, Germany). TRIS-Base was from Roche (Basel, Switzerland). Velocity DNA polymerase was from Bioline (London, UK). Elk milk powder was from Campina (Zaltbommel, The Netherlands). Rabbit-anti-RNF4 (Eurogentec, custom made, Vyas et al., 2013), HRP-conjugated Donkey-anti-Rabbit secondary antibody was from Thermo Fisher Scientific. Western Bright Quantum Western blotting detection kit was from Advansta (Menlo Park, CA, USA).

### Generation of the TULIP2 Toolbox

For the construction of the TULIP2 plasmids, using the previous TULIP plasmid (Kumar et al., 2017), a 1.7 Kbp fragment was amplified by PCR with Velocity DNA polymerase using either FW-NheI-H-TULIP2: AGCTAGCATGCATCACCATCATCACCACCACCACCATCACCAATCAACAAGTTTGTACAAAAAAGCTGAACGor FW-NheI-HF-TULIP2: AGCTAGCATGCATCACCATCATCACCACCACCACCATCACGATTACAAGGATGACGACGATAAGCAATCAACAAGTTTGTACAAAAAAGCTGAACG as forward primer for H-TULIP2 and HF-TULIP2, respectively, and RV-TULIP2: AGAATTCCGGATGAGCATTCATCAGG as reverse. PCR fragment was digested with NheI and AgeI restriction enzymes and cloned between the NheI and AgeI sites within the TULIP plasmids.

### Generation of TULIP2 Lentiviral Plasmids

**Figure 2:**
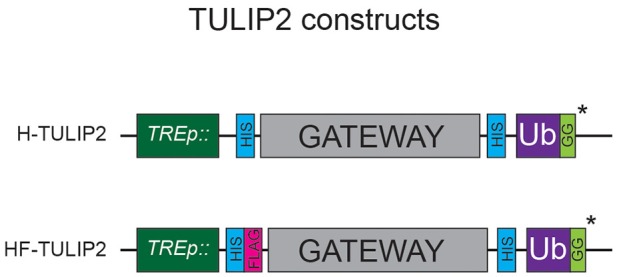
TULIP2 Constructs. Schematic representation of the TULIP2 cloning cassette including the *TRE* promoter, 10xHIS and tandem 10xHIS-FLAG tag, Gateway cloning cassette, linker containing 10xHIS and active ubiquitin. *ΔGG constructs lack the C-terminal GG motif.

TULIP2 plasmids are generated by Gateway® cloning (Thermo Fisher Scientific) according to vendor instructions. LR reactions are performed using a donor plasmid containing an E3 enzyme cDNA without stop codon and a TULIP2 plasmid (Figure 2) as destination vector. cDNAs from several E3 enzymes without stop codon can be obtained from repositories such as DNASU (Seiler et al., 2014) or the CCSB Human ORFeome Project (Lamesch et al., 2007). Additionally, cDNAs can also be subcloned into donor vectors by Gateway® cloning BP reactions (Thermo Fisher Scientific). In this article, we use pDONR207-RNF4, which was previously described (Kumar et al., 2017).

### Cell Culture

293T and U2OS were cultured in Dulbecco's modified Eagle's medium (DMEM) supplemented with 10% Fetal Bovine Serum (FBS) and 100 U/mL penicillin/100 μg/mL streptomycin at 37°C and 5% CO2 unless specifically specified. The cells were regularly tested for mycoplasma contamination.

### TULIP2 Lentivirus Production

293T cells were seeded at 30% confluency in a T175 flask containing 16 mL of DMEM + 10% FBS and allowed to attach overnight. Next, a 2 mL transfection mixture was prepared in 150 mM NaCl containing 7.5 μg pMD2.G (#12259, Addgene), 11.4 μg pMDLg-RRE (#12251, Addgene), 5.4 μg pRSV-REV (#12253, Addgene), 13.7 μg TULIP2 plasmid and 114 μL of 1 mg/mL Polyethylenimine (PEI) solution. All the components were mixed by vortexing and incubated 10 min at room temperature. Subsequently, the transfection mix was added to the cells. The day after transfection, culture medium was replaced by fresh DMEM/FBS/Pen/Strep. Three days after transfection, lentiviral suspension was filtered by passing through a 0.45 μm syringe filter (PN4184, Pall Corporation). Lentiviral particle concentration was determined using the HIV Type 1 p24 antigen ELISA Kit (ZeptoMetrix Corporation).

### TULIP2 Cell Lines

U2OS cells were seeded in 15 cm diameter plates at 10% confluency (2 × 10^6^ cells) and allowed to attach overnight. Next day, cell culture medium was replaced with cell culture medium containing 3.2 μg of lentiviral particles and polybrene 8 μg/mL final concentration. Twenty-four hours later, medium was replaced with fresh medium. Three days after lentiviral transduction, TULIP2 construct-positive clones were selected by adding puromycin 3 μg/mL to the culture medium.

### Purification of TULIP2 Conjugates

**Figure 3:**
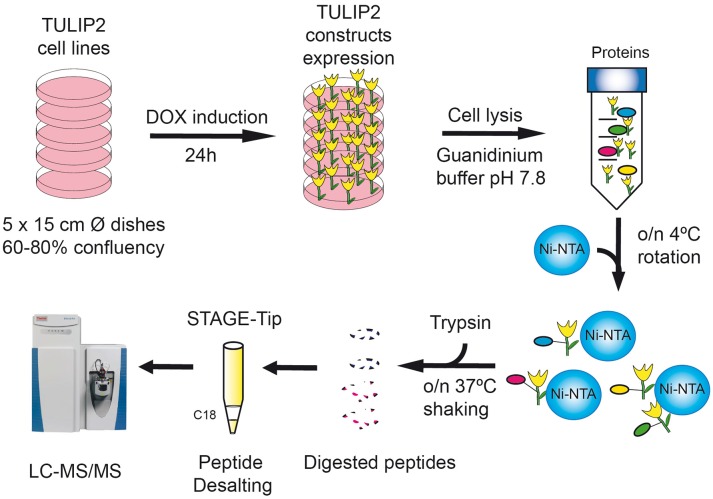
TULIP2 methodology overview. Cells stably containing the E3-TULIP2 expression cassettes are cultured up to 60–80% confluency. The expression of the E3-TULIP2 constructs is induced for 24 h and then they are lysed in Guanidinium buffer and incubated overnight with Ni-NTA beads. Subsequently, beads are washed with different washing buffers and on-the-beads digestion of TULIP2 conjugates with trypsin is performed overnight at 37°C while shaking. Next, digested peptides are desalted by C18 STAGE-Tipping and analyzed by LC-MS/MS.

A method overview of TULIP2 methodology is provided in Figure 3. Five 15 cm diameter plates of U2OS cells were grown up to 60–80% confluence and the expression of TULIP2 construct was induced with 1 μg/mL doxycycline for 24 h. Next, cells were treated for 5 h with proteasome inhibitor MG132 (Sigma Aldrich) at 10 μM. Subsequently, cells were washed twice with ice-cold PBS, scraped and transferred to a 50 mL tube. Cells were spun down 5 min at 500 × g, supernatant was discarded and cells were transferred to a 15 mL tube with 5 mL PBS. At this point, a 100 μL aliquot was taken to serve as input sample. After spinning down 1 min at 500 x g and discarding supernatant, input sample cells were lysed in 100 μL SNTBS buffer (2% SDS, 1% NP-40, 50 mM TRIS pH 7.5, 150 mM NaCl). Rest of the sample was centrifuged 3 min at 500 × g and the supernatant discarded.

Cell pellet was lysed in 10 mL Guanidinium buffer (6M guanidine-HCl, 0.1M Sodium Phosphate, 10 mM TRIS, pH 7.8). Samples were homogenized at room temperature by sonication using a tip sonicator (Q125 Sonicator, QSonica, Newtown, USA). Sonication was performed at 80% amplitude during 5 s. Subsequently, protein concentration was determined by BiCinchoninic Acid (BCA) Protein Assay Reagent (Thermo Scientific) and sample total protein content was equalized accordingly.

Lysates were supplemented with 5 mM β-mercaptoethanol and 50 mM Imidazole pH 8.0. 100 μL of nickel-nitrilotriacetic acid-agarose (Ni-NTA) beads (QIAGEN), were equilibrated with Guanidinium buffer supplemented with 5 mM β-mercaptoethanol and 50 mM Imidazole pH 8.0, added to the cell lysates and incubated overnight at 4°C under rotation.

After lysate-beads incubation, samples were centrifuged 5 min at 500 × g and the supernatant was discarded. Ni-NTA beads were transferred with 1 mL Wash buffer 1 (6 M Guanidine-HCl, 0.1 M Sodium Phosphate, 10 mM TRIS, 10 mM Imidazole, 5 mM β-mercaptoethanol, 0.2% Triton X-100, pH 7.8) to an Eppendorf LoBind tube (Eppendorf). Centrifuged again, supernatant discarded, and moved to a new LoBind tube with Wash buffer 2 (8 M Urea, 0.1 M Sodium Phosphate, 10 mM TRIS, 10 mM imidazole, 5 mM β-mercaptoethanol, pH 8). Same procedure was repeated with Wash buffer 3 (8 M urea, 0.1 M Sodium Phosphate, 10 mM TRIS, 10 mM imidazole, 5 mM β-mercaptoethanol, pH 6.3). Next, beads were washed twice with Wash buffer 4 (8 M urea, 0.1 M Sodium Phosphate, 10 mM TRIS, 5 mM β-mercaptoethanol, pH 6.3). In every wash step, beads were allowed to equilibrate with the buffer for 15 min under rotation.

The steps for the purification of the TULIP2 conjugates are indicated in a simplified manner in (Supplementary Protocol 1).

### Trypsin Digestion

After second wash with Wash buffer 4, Ni-NTA beads were separated from the buffer by passing through a 0.45 μm filter Ultrafree-MC-HV spin column (Merck-Millipore) which had been previously equilibrated with 250 μL of ABC buffer (50 mM ammonium bicarbonate). Using 400 μL of ABC buffer, Ni-NTA beads were transferred to a new Eppendorf LoBind tube and 500 ng of sequencing grade modified trypsin (Promega) were added to the ABC buffer-beads suspension. Digestion was performed overnight at 37°C while shaking at 1,400 rpm.

### Electrophoresis and Immunoblotting

0.1% of the whole-cell extract (Inputs) and 5% of the HIS-purified proteins (TULIP and TULIP2 conjugates) were separated on Novex 4–12% gradient gels (Thermo Fisher Scientific) using NuPAGE® MOPS SDS running buffer (50 mM MOPS, 50 mM TRIS-base, 0.1% SDS, 1 mM EDTA pH 7.7) and transferred onto Amersham Protran Premium 0.45 NC Nitrocellulose blotting membrane (GE Healthcare) using a Bolt Mini-Gel system (Thermo Fisher Scientific), which was used for both the gel electrophoresis and the protein transfer to the membrane according to vendor instructions.

Membrane was stained with Ponceau-S (Sigma Aldrich) to determine total amount of protein loaded. Next membrane was de-stained with PBS + 0.1% Tween-20 and, subsequently, was blocked with Blocking solution (8% Elk milk, 0.1% Tween-20 in PBS) for 1 h. Next, membrane was incubated overnight with 2 ml of a 1:2500 dilution of anti-RNF4 antibody in blocking solution. Next day, membranes were washed 3 times 10 min with PBS + 0.1% Tween-20. Subsequently, membranes were incubated for 1 h with a 1:5000 dilution of HRP-conjugated Donkey-anti-rabbit secondary antibody in blocking solution and washed another 3 times 10 min with PBS+0.1% Tween 20.

Chemiluminescence reaction was initiated with Western Bright Quantum Western blotting detection kit and measured in a ChemiDoc^TM^ imaging system (BIO-RAD, Hercules, CA, USA). The quantification of the signal corresponding to the TULIP and TULIP2 constructs was done using FIJI software (Schindelin et al., 2012).

### Mass Spectrometry Sample Preparation

Trypsin-digested peptides were separated from the beads by filtering through a 0.45 μm filter Ultrafree-MC-HV spin column (Merck-Millipore) which had been previously equilibrated with 250 μL of ABC buffer. Flow through was collected in an Eppendorf LoBind tube and acidified by adding 2% TriFlourAcetic (TFA) acid. Subsequently, peptides were desalted and concentrated on STAGE-Tips as previously described (Rappsilber et al., 2007). STAGE-Tips were in-house assembled using 200 μL micro pipet tips and a C18 matrix. STAGE-Tips were activated by passing through 100 μL of methanol. Subsequently 100 μL of Buffer B (80% acetonitrile, 0.1% formic acid), 100 μL of Buffer A (0.1% formic acid), the peptide sample, and two times 100 μL Buffer A were passed through the STAGE-tip. Elution was performed in 50 μL of 50% acetonitrile, 0.1% formic acid.

Samples were vacuum dried using a SpeedVac RC10.10 (Jouan, France) and stored at −20°C. Prior to mass spectrometry analysis, samples were reconstituted in 10 μL 0.1% Formic acid and transferred to autoload vials.

### LC-MS/MS

All the experiments were performed on an EASY-nLC 1000 system (Proxeon, Odense, Denmark) connected to a Q-Exactive Orbitrap (Thermo Fisher Scientific, Germany) through a nano-electrospray ion source. The Q-Exactive was coupled to a 25 cm silica emitter (FS360-75-15-N-5-C25, NewObjective, Woburn, MA, USA) packed in house with 1.9 μm C18-AQ beads (Reprospher-DE, Pur, Dr. Manish, Ammerbuch-Entringen, Germany).

Twenty percent of the sample was injected in a 100 min chromatography gradient from 0 to 30% acetonitrile and then increasing to 95% acetonitrile prior to column re-equilibration with flow rate of 200 nL/min. The mass spectrometer was operated in a Data-Dependent Acquisition (DDA) mode with a top-10 method and a scan range of 300–1,600 m/z. Full-scan MS spectra were acquired at a target value of 3 × 10^6^ and a resolution of 70,000, and the Higher-Collisional Dissociation (HCD) tandem mass spectra (MS/MS) were recorded at a target value of 1 × 10^5^ and with a resolution of 17,500, an isolation window of 2.2 m/z, and a normalized collision energy (NCE) of 25%. The minimum AGC target was 1 ×10^4^. The maximum MS1 and MS2 injection times were 250 and 60 ms, respectively.

The precursor ion masses of scanned ions were dynamically excluded (DE) from MS/MS analysis for 20 s. Ions with charge 1, and >6, were excluded from triggering MS2 analysis.

### Mass Spectrometry Data Analysis

All raw data were analyzed using MaxQuant (version 1.6.7.0) as described previously (Tyanova et al., 2016a). We performed the search against an *in silico* digested UniProt reference proteome for Homo sapiens including canonical and isoform sequences (27th May 2019). Database searches were performed according to standard settings with the following modifications. Digestion with Trypsin/P was used, allowing 4 missed cleavages. Oxidation (M), Acetyl (Protein N-term), and GlyGly (for ubiquitination sites) were allowed as variable modifications with a maximum number of 3. Carbamidomethyl (C) was disabled as a fixed modification. Label-Free Quantification was enabled, not allowing Fast LFQ. All peptides were used for protein quantification.

Output from MaxQuant Data were exported and processed in MS Excel for further filtering, processing of the data, and visualization.

For the statistical analysis of RNF4-TULIP2 samples, output from the analysis in MaxQuant was further processed in the Perseus computational platform (v 1.6.7.0) (Tyanova et al., 2016b). LFQ intensity values were log2 transformed. Potential contaminants and proteins identified by site only or reverse peptide were removed. Samples were grouped in experimental categories and proteins not identified in 3 out of 3 replicates in at least one group were also removed. Missing values were imputed using normally distributed values with a 1.8 downshift (log2) and a randomized 0.3 width (log2) considering whole matrix values. Statistical analysis was performed to determine which proteins were significantly enriched in the wild type RNF4 samples compared to the ΔGG samples (*t*-test with permutation-based False Discovery Rate (FDR) = 0.05 and S0 = 0.1).

## Results

### TULIP vs. TULIP2

Previously, TULIP methodology was employed to identify the SUMO Targeted Ubiquitin Ligase (STUbL) RNF4 specific ubiquitination targets (Kumar et al., 2017). In order to compare the new TULIP2 methodology with the previous TULIP methodology version, we cloned the RNF4 into the H-TULIP2 plasmids. Next, we generated lentiviral particles containing the RNF4-TULIP2 constructs and used them to stably introduce the RNF4-TULIP2 constructs in U2OS cells by lentiviral transduction. Positive clones were selected with puromycin.

**Figure 4:**
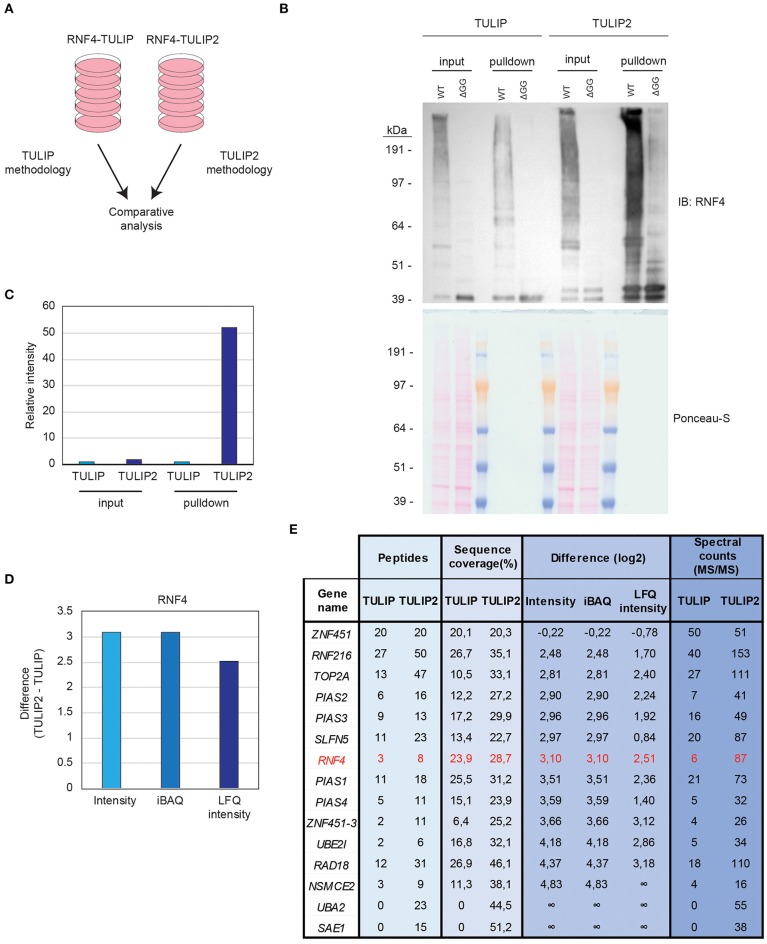
TULIP vs. TULIP2. **(A)** Experimental design to compare TULIP vs. TULIP2. **(B)**. U2OS cells containing either RNF4-TULIP or RNF4-TULIP2 expression cassettes were induced overnight with doxycycline, lysed and TULIP/TULIP2 conjugates purified according to TULIP or TULIP2 methodology, respectively. The efficiency of the expression and the purification was analyzed by immunoblotting. Ponceau-S is provided as loading control. **(C)** Quantification of the intensity from the immunoblotting analysis performed in **(B)**. Intensity of the signal in TULIP samples is normalized as 1. **(D)** Graph depicting the log2 difference between RNF4-TULIP2 and RNF4-TULIP samples for RNF4 after mass spectrometry analysis in terms of Intensity, iBAQ or LFQ intensity. **(E)** Table indicating the values for number of peptides, sequence coverage, log2 difference of intensities after LC-MS/MS analysis and spectral counts of top RNF4-specific ubiquitination targets comparing RNF4-TULIP and RNF4-TULIP2 samples.

Cells expressing RNF4-TULIP and RNF4-TULIP2 constructs were grown in equal amount, induced for the same time and treated for 5 h with the proteasome inhibitor MG132. Next, cells were lysed and the RNF4-TULIP and RNF4-TULIP2 conjugates were purified in parallel following the TULIP methodology protocol (Gonzalez-Prieto and Vertegaal, 2019) or the TULIP2 method introduced in this article, respectively (Figure 4A). Next, whole cell extracts and 5% of the HIS-pulldown samples were analyzed by immunoblotting using an anti-RNF4 antibody (Figure 4B). While the RNF4-TULIP2 constructs were expressed relatively higher than their RNF4-TULIP counterparts by a factor of 1.7, the amount of RN4-TULIP2 conjugates purified were 52.2 times higher compared to the amount of RNF4-TULIP conjugates while using the same amount of starting material (Figure 4C).

Next, we decided to perform a comparison using three biological replicates of RNF4-TULIP2 samples and the RNF4-TULIP samples from Kumar et al. (2017) both generated after treating with the proteasome inhibitor MG132. In both cases, 20% of the RNF4-TULIP or RNF4-TULIP2 samples were injected in the mass spectrometer and analyzed using the same chromatography gradients. All three biological replicates of each sample set were grouped together for performing comparisons. Signal corresponding to RNF4 was more than 8 times higher in the TULIP2 samples compared to TULIP samples when looking at Intensity or iBAQ MaxQuant output values and more than 5 times in the case of the values of the Label Free Quantification intensity (Figure 4D).

Previously, using TULIP methodology, we identified components of the sumoylation machinery and other proteins such as TOP2A, SLFN5, RAD18, and RNF216 as the most important SIM- and MG132-dependent RNF4 targets. Using TULIP2 methodology we were able to increase the number of peptides, the percentage of sequence coverage, intensity, iBAQ, and LFQ intensity values and the number of spectral counts for all these RNF4 direct ubiquitination targets (Figure 4E, Supplementary Dataset 1).

While TULIP methodology allowed us to identify SUMO E3s and E2 as ubiquitination targets for RNF4, TULIP2 methodology also identified the SUMO E1 enzyme (SAE1/UBA2) as an RNF4 ubiquitination target, indicating that, upon SUMOylation, all the members of the SUMOylation machinery, including E1, E2, and E3 enzymes, are targeted for degradation in an RNF4-dependent manner.

**Figure 5:**
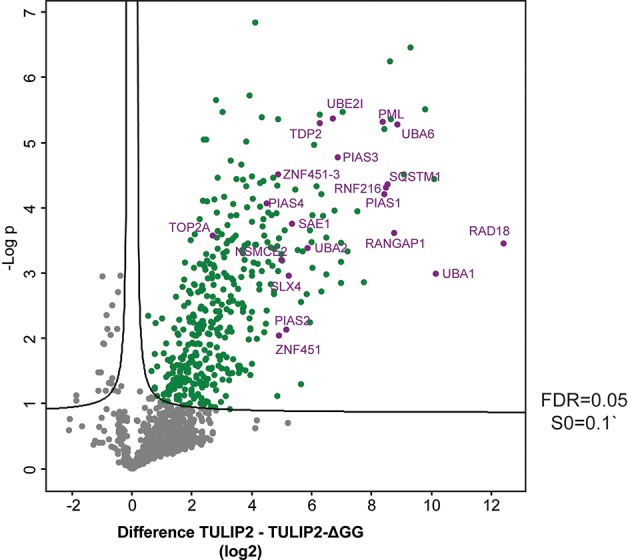
RNF4-TULIP2 ubiquitination targets. Volcano plot depicting RNF4-TULIP2 conjugates comparing to RNF4-TULIP2-ΔGG samples. Each dot represents a protein. Green dots represent proteins that are statistically enriched in the RNF4-TULIP2 samples compared to RNF4-TULIP2-ΔGG samples for an FDR = 0.05 and S0 = 0.1. Purple labeled dots represent proteins relates to the SUMOylation machinery or top main ubiquitination targets previously identified by TULIP methodology.

Next, in order to generate a new list of RNF4 ubiquitination targets by using TULIP2 methodology, we performed a second analysis including RNF4-TULIP2 samples and RNF4-TULIP2-ΔGG samples as negative control. We performed 3 biological replicates of each construct in order to perform statistical comparisons. Comparison between the RNF4-TULIP2 and RNF4-TULIP2-ΔGG identified 409 RNF4-TULIP2 conjugated proteins (Figure 5, Supplementary Dataset 2). Moreover, mass spectrometry analysis also allowed to identify 372 specific ubiquitination sites in 209 proteins (Supplementary Dataset 3), including many members of the sumoylation machinery and the previously identified as main ubiquitination targets targeted for degradation by RNF4 in a SUMO-dependent manner.

## Discussion, Advantages, and Pitfalls

In this article we have performed a comparison between our previously published TULIP methodology (Kumar et al., 2017) and an improved version, which we have termed TULIP2 methodology. Compared to previous version, for the STUbL RNF4, it achieves a more than 50 times improvement in terms of purification efficiency (Figures 4B,C). This methodology can be implemented in any laboratory interested in the identification of the ubiquitination targets of a given E3 of interest. Furthermore, the simplification of the protocol by suppressing the elution and size exclusion filter-based sample concentration results in a reduction of the execution costs of the experiments. Moreover, the introduction of the HIS-FLAG TULIP2 plasmids allow the employment of an anti-FLAG tag antibody when a good specific antibody for immunoblotting is not available for the E3 enzyme of interest or for unambiguous identification respect of the endogenous E3 enzyme. Together, all these improvements enable the implementation of the TULIP2 methodology in any research group with access to a mass-spectrometry facility. To facilitate the implementation of the TULIP2 methodology in any laboratory we have included an annotated step-by-step protocol from the induction of the expression of the TULIP2 constructs until the isolation of the trypsin-digested peptides corresponding to the TULIP2 constructs and conjugates.

The improvement achieved by TULIP2 allowed us not only to have a better coverage of the RNF4 ubiquitination targets after mass spectrometry analysis, but also to identify new RNF4 ubiquitination substrates (Figures 4E, 5, Supplementary Datasets 1, 2). Moreover, we could determine the specific ubiquitination sites of many of the identified RNF4 targets (Supplementary Dataset 3). While previous TULIP methodology allowed us to identify 31 ubiquitination sites on 16 proteins (Kumar et al., 2017), these numbers increased to 372 and 209, respectively, using TULIP2 methodology.

The improvement achieved by TULIP2 methodology facilitates the identification of specific substrates for other E3 enzymes which are less stable, their ubiquitination targets less abundant and/or have a lower ubiquitination activity than RNF4. The identification of the E3-specific ubiquitination substrates using TULIP methodology was still challenging and very large amounts of cells needed to be lysed to obtain the minimum amounts of TULIP conjugates to allow identification by mass spectrometry. TULIP2 methodology solves this major drawback. TULIP2 is straightforward and enables the systematic identification of the specific ubiquitination targets of virtually every HECT- and RING-type E3 enzyme. Using Gateway cloning, any E3-ligase cDNA can be cloned into the TULIP2 plasmids.

Nevertheless, the TULIP2 methodology still shares some limitations with the previous version of the method (Kumar et al., 2017). Some E3-TULIP2 constructs might not be functional due to steric hindrance and the size of the E3 to be cloned into the TULIP2 plasmids is limited by the capacity of the lentiviral particles. As an indication, we have been able to clone E3 enzymes with cDNA sizes up to 6 kilobase pairs. Some E3-TULIP2 constructs might be very rapidly targeted for degradation by the proteasome via autoubiquitination given that the already present ubiquitin moiety is a signal for ubiquitin chain lengthening. Thus, inhibition of the proteasome might be required to be able to purify sufficient amount of TULIP2 conjugates to secure identification by mass spectrometry.

It is also worth noting that, although TULIP2-attached E3s represent a bulky tag that hamper the utilization of the attached ubiquitin by other E3s to ubiquitinate their targets, potentially ubiquitin moieties from the TULIP2 constructs can still be used by other E3s. Thus, including catalytically dead mutants of the E3s of interest as an additional negative control to the ΔGG TULIP2 constructs might be advantageous. Finally, the probability of success in identifying the specific ubiquitination substrates for a given E3 enzyme highly depends on the sensitivity of the mass spectrometry equipment employed and the amount of sample injected. The signal corresponding to the TULIP2 conjugates is commonly below the signal corresponding to the common unspecific binders to Ni-NTA beads, making good enrichment is critical for successful identification.

## Data Availability Statement

TULIP2 construct plasmids are freely available from the González-Prieto lab upon reasonable request.

The mass spectrometry proteomics data have been deposited to the ProteomeXchange Consortium via the PRIDE (Perez-Riverol et al., 2019) partner repository with the dataset identifier PXD015437.

## Author Contributions

RG-P designed and constructed the TULIP2 plasmids. RG-P, DS-L, and GA performed experiments. GA was supervised by DS-L. RG-P and DS-L made the figures. RG-P wrote the manuscript with input from DS-L. All authors contributed to manuscript revision, read and approved the submitted version.

### Conflict of Interest

The authors declare that the research was conducted in the absence of any commercial or financial relationships that could be construed as a potential conflict of interest.

## Supplementary Material

Supplementary Dataset 1 Values from the mass spectrometry analysis comparing RNF4-TULIP and RNF4-TULIP2.

Supplementary Dataset 2 Statistical analysis of RNF4-TULIP2 samples compared to RNF4-TULIP2-ΔGG samples.

Supplementary Dataset 3 Specific ubiquitination sites identified by mass spectrometry in RNF4-TULIP2 samples.

Supplementary Protocol 1 TULIP2 benchtop step-by-step protocol.
