# Supplementary material for: TULIP2: An Improved Method for the Identification of Ubiquitin E3-Specific Targets
Source: Front Chem. 2019 Nov 26;7:802. doi: 10.3389/fchem.2019.00802 (PMC6901917; doi:10.3389/fchem.2019.00802)
Supplement: Supplementary Protocol 1 — TULIP2 benchtop step-by-step protocol. [file Presentation_1.pdf]

TULIP2 benchtop Step-by-step protocol

General mass spectrometry sample preparation guidelines
In general, it is pivotal to avoid detergent and Poly-Ethylene Glycol (PEG) contamination. Micropipettes and glassware cleaned in general facilities tend to be contaminated with traces of commonly used detergents (namely, Triton X-100, NP-40, Tween-20, SDS, etc.). To avoid detergent contamination, it is advised to rinse thoroughly every piece of glassware and micropipettes with molecular biology grade water and methanol. DO NOT USE Syringes to filter buffers as the embolus is usually lubricated with PEG.

Day 0: Add doxycycline 24h prior to lysis for induction of TULIP2 construct expression.

Previously, perform a doxycycline concentration titration to adjust the expression levels of your TULIP2 constructs to close-to-endogenous levels for your E3 enzyme of interest.

Day 1: Lysis of cells (5x 15cm confluent dishes)

The volumes indicated here are for 5 x 15 cm confluent dishes, if a different amount of cells is employed scale up or down the volumes accordingly.

- Before lysis, add any treatment to cells if required.
- Remove cell culture medium.
- Wash the cells dishes 2x with 10mL ice-cold PBS.
- Remove the 2nd wash entirely.
- Add 2 mL ice-cold PBS per dish, scrape cells, and collect them in 50 mL tubes.
- Spin down cells (500 x g, 5 min, 4°C).
- Remove PBS and resuspend the cell pellet in 5 mL ice-cold PBS
- Transfer an aliquot (5% of the sample) to a 1.5 mL tube to serve as input sample.
  For the Input sample:
    - Spin down cells (500 x g, 1min)
    - Remove supernatant and lyse cells with 100 µL SNTBS buffer.
    - Mix by vortexing and snap freeze in liquid nitrogen.
- Transfer the cell suspension to a 15 mL tube
- Spin down the main batch of cells (500 x g, 3 min)
- Remove supernatant.
- Lyse the cells in 10 mL of Lysis Buffer (pH 7.8) while vortexing.
  The lysate should be mostly clear, without any floating debris. Agitate thoroughly to complete lysis

At this point, lysates can be snap frozen in liquid nitrogen and stored for an indefinite period of time. This is advantageous to accumulate different biological repeats of a given condition and then process them simultaneously to purify the TULIP2 conjugates.

Day 2: Ni-NTA HIS purification (I)

- Thaw the lysate.
- Sonicate the lysates for 5 seconds at 80% amplitude using a microtip sonicator.
- Repeat the sonication step twice and mix the samples between sonication steps.
- Equalize all lysates using BCA (or Bradford).
- Add to the lysates: imidazole 50 mM, pH 8 and 5 mM β-mercaptoethanol (final concentration)
- Mix the lysates by rotation while equilibrating Ni-NTA beads.
- Prepare 10 µL Ni-NTA beads (dry volume) per 1 mL of lysate.
- Wash Ni-NTA beads 4 times with Lysis Buffer supplemented with 50 mM imidazole pH 8 and 5 mM β-mercaptoethanol.

- Add the Ni-NTA beads to the lysates (10 µL beads / 1 mL lysate).
- Incubate overnight at 4°C mixing by rotation.

Day 3: Ni-NTA HIS purification (II) and digestion on the beads

- Spin down the beads at 500 x g, 2 minutes.
- Remove all supernatant.
- Transfer beads to a Lo-Bind Eppendorf tubes with 1 mL of Wash buffer 1
  Wash buffer volume should be at least 5 times the bead volume in every wash.
- Pellet beads briefly at 500 x g, 1min
- Remove supernatant completely after every wash.
- Wash with Wash Buffer 2 and change tubes afterwards.
  Wash buffer 1 contains Triton X-100, although it is important to avoid unspecific interactions with beads, it is an inconvenient for mass spectrometry analysis. Changing tubes between washes minimizes the amount of Triton X-100 contamination.
- Wash with Wash Buffer 3. Perform this wash while rotating for 15 minutes and change tubes afterwards.
- Wash with Wash Buffer 4. Perform this wash while rotating for 15 minutes.
- Repeat wash with Wash Buffer 4. Perform this wash while rotating for 15 minutes.
- Transfer the beads to a pre-washed (with freshly prepared ABC buffer) 0.45 µm filter column and spin down at 10,000 x g 10min to separate the beads from the buffer.
- Take the beads with 400µL ABC (in two times with 200µL) and place them in a LoBind epp tube.
- A small fraction of beads (10%) should be kept for analysis by immunoblotting **(Pull Down)**
- Add 500ng Trypsin to each sample.
- Incubate overnight in a bench shaker at 37°C and 1,400 rpm.

Day 4: Desalting by Stage Tip and Mass Spectrometry preparation

- After digestion, spin down the samples at 8,000 x g, 10min in a pre-washed (with fresh ABC 50mM 200µL 10min at 8,000 x g) 0.45 µm filter column, collect the digested proteins in a LoBind eppendorf tube and remove the filter with beads.
- Desalt peptides according to the Stage Tip protocol (Rappsilber et al., 2007).
- Perform the elution from the stagetips with 40% Acetonitrile, 0.1% Formic Acid.
- Lyophilize peptides.
- Resuspend in 0.1% Formic acid.
- Transfer to autoload vials.
- Analyze by Mass Spectrometry.

<u>**Annex I: His-pulldown buffers**</u>

SNTBS: 2% SDS, 1% NP-40, 50mM Tris, 150 mM NaCl, pH 7.5.
Lysis Buffer: 6 M guanidine-HCl, 0.1M Sodium Phosphate, 10 mM Tris-HCl, pH 7.8.
Wash Buffer 1: 6 M Guanidine-HCL, 0.1M Sodium Phosphate, 10 mM Tris, 10 mM Imidazole, 5 mM β-mercaptoethanol, 0,2 % Triton X-100, pH 7.8
Wash buffer 2: 8 M Urea, 0.1M Sodium Phosphate, 10 mM Tris, 10 mM imidazole, 5mM β-mercaptoethanol, pH 8
Wash buffer 3: 8 M Urea, 0.1M Sodium Phosphate, 10 mM Tris, 10 mM imidazole, 5 mM β-mercaptoethanol, pH 6.3
Wash buffer 4: 8 M urea, 0.1M Sodium Phosphate, 10 mM Tris, 5 mM β-mercaptoethanol, pH 6.3.
ABC buffer: 50 mM Ammonium bicarbonate, pH 8

Annex II: Wash buffer recipes from Stock solutions

To make 10 mL **Wash Buffer 1**, add 20 µL 5 M imidazole **pH 8.0**, 3.5 µL β-ME and 100 µL 20% Triton X-100 to 10 mL Lysis Buffer.

To make 10 mL **Wash Buffers 2, 3 and 4.**

Stocks	**Wash 2** [10mL]	**Wash 3** [10mL]	**Wash 4** [10mL]
1M Tris pH **8.0**	0.1 mL		
1M Tris pH **6.3**		0.1 mL	0.1 mL
1M Tris pH **7.0**			
1M $\underline{\mathbf{Na_2}}HPO_4$	932 µL	216 µL	216 µL
1M $Na\underline{\mathbf{H_2}}PO_4$	68 µL	784 µL	784 µL
9M urea [fresh]	8.88 mL	8.88 mL	8.88 mL
5M imidazole	20 µL (**pH 8**)	20 µL (**pH 7**)	
14.3M β-ME	3.5 µL	3.5 µL	3.5 µL

Prepare the 9 M urea stock fresh on the same day as the pulldown.
1M Na_2HPO_4 crashes at room temperature, store at 37°C.
